# Prognostic value of Linc00662/miR-16-5p/FASN in cervical cancer and regulation of tumor progression

**DOI:** 10.1186/s41065-025-00520-6

**Published:** 2025-08-05

**Authors:** Yu Yang, Zhanping Guo

**Affiliations:** Department of East District Laboratory, Xinxiang Central Hospital, Northwest corner of the intersection of East Rongxiao Road and Dongming Avenue, Muye District, Xinxiang City, Henan Province 453001 China

**Keywords:** Cervical cancer, Linc00662, MicroRNA-16-5p, Fatty Acid Synthase

## Abstract

**Background:**

Cervical cancer (CC) is the world's single most frequent gynecological cancer, is more than 500,000 new annual cases globally, and is a serious threat to women's reproductive health. LncRNAs have significant effects on human diseases; nevertheless, the expression of Linc00662 in CC and its mechanism of action are not yet entirely clear. The goal of the work was to investigate the expression, prognostic value and biological utility of Linc00662 in CC progression and to identify its underlying mechanisms in molecular terms.

**Methods:**

Expression levels of Linc00662, miR-16-5p and FASN in CC tissues and cells were detected through real-time quantitative PCR. Determination of cell proliferative capacity by CCK-8. Cell migration and invasion were assessed through Transwell assay. Binding of Linc00662 to miR-16-5p was mediated through a dual-luciferase reporter gene test was validated.

**Results:**

Linc00662 expression levels were significantly elevated in CC. High Linc00662 expression was strongly linked to increased tumor size, later FIGO staging, poorer tumor differentiation, mesenchymal infiltration, and lymph node metastasis, and high Linc00662 expression predicted a poor prognosis. Silencing Linc00662 reduced the proliferation, migration, and invasion of CC cells. Furthermore, Linc00662 negatively regulated miR-16-5p and indirectly regulated the upregulation of FASN expression.

**Conclusions:**

Linc00662 positively regulates FASN expression through targeting miR-16-5p and facilitates CC cell proliferation, migration and invasion, promoting CC progression.

## Introduction

Cervical cancer (CC) is one of the most common gynecological cancers in the world, with more than 500,000 new cases arising globally each year [1, 2]. In recent years, in China, the morbidity of cervical cancer has shown a rising trend and the age of onset has also demonstrated a younger age, which seriously endangers women's reproductive health [3]. In the past, researchers have developed various methods for cancer treatment, including surgery, radiation therapy, chemotherapy, hormone therapy, and targeted therapies that address genetic alterations and associated signaling pathways. While many of these methods are still in use today, there remains a significant gap in effective clinical treatment options [4]. Existing scholars have proposed that targeting key components of the tumor microenvironment (TME) is a crucial strategy in cancer treatment. This integrated approach, which focuses on multiple elements of the TME, can disrupt its pro-tumor dynamics and improve treatment outcomes [5]. Tumor cells constitute the primary component of TME, and directly targeting these cells remains a fundamental aspect of cancer treatment. Consequently, it is essential to conduct comprehensive research on CC cells and to identify novel biomarkers and therapeutic targets.

Long non-coding RNAs (LncRNAs) are a subset of transcripts above 200 nucleotides in length that encode no proteins [6]. LncRNAs were initially called genomic transcriptional “rubbish”, but now more and more works have evidenced that LncRNAs have a significant effect in human diseases and are significantly correlated with the development of various tumors [7–10]. Linc00662 is involved in the development of breast cancer, bladder cancer, gallbladder cancer and other tumors [11–13]. However, the expression and mechanism of Linc00662 in CC are not clear.

MicroRNAs (miRNAs) are a class of non-coding single-stranded RNAs encoded by endogenous genes, which are key regulators that inhibit the expression of target genes [14, 15]. miRNAs also have a major regulatory effect in CC pathogenesis [16]. miR-16-5p is involved in CC progression and is lowly expressed in CC cells [17]. However, there are fewer studies on the involvement of miR-16-5p in CC progression, and further studies on the role of miR-16-5p in CC are still needed.

Fatty acid synthase (FASN) is an enzyme protein with a specific structure and function that plays a key role in catalyzing fatty acid synthesis. FASN has been linked to the advancement of various cancers, including hepatocellular carcinoma, rectal carcinoma and breast cancer [18–20]. In CC, FASN is considered a primary diagnostic and curative target [21, 22]. However, the effects of FASN in CC still need to be further investigated.

The investigation of Linc00662 in CC has thus far identified only two pathways: Linc00662/miR-103a-3p/PDK4 and Linc00662/miR-497-5p/CDC25A [23, 24]. These findings are relatively limited in scope. The current work was to investigate the expression, prognostic value and biological utility of Linc00662 in CC, and to confirm its role in the regulation of miR-16-5p and FASN. This will contribute to the theoretical rationale for future diagnosis and treatment in CC.

## Methods and materials

### Study subjects

The work abided by the Declaration of Helsinki and was supported through the Medical Ethics Committee of Xinxiang Central Hospital. 118 Patients were recruited at Xinxiang Central Hospital and each subject signed an informed consent form.

### Sample collection

Tissue samples collected during surgery included tumor and normal paracancerous tissue that met the requirements for testing. The collected tissues were identified and sorted by two pathologists. The Organisations were labeled, chilled in water nitrogen and stored at -80 °C.

### Cell culture and transfection

Human-derived CC cell lines (C33A, HeLa, HT-3, and SiHa) and normal cervical epithelial cells (HCE 16/3) were obtained from ATCC. Cells were stored in DMEM medium and cultured at 37 °C with 5% CO2. Cells were transfected with Lipofectamine 2000 reagent as required and according to the instructions.

### Real-time quantitative PCR

RT-qPCR assays were conducted as previously described [25]. Total RNA was extracted using the Fast Pure Viral DNA/RNA Mini Kit (Vazyme, Nanjing, China). According to the instructions for using SYBR Green Realtime PCR Master (Vazyme, Nanjing, China), GAPDH and U6 served as internal controls and were placed in Light Cycler ®96 PCR instrument, and the data were obtained by the 2^−ΔΔCt^ method after obtaining the results.

### Cell proliferation

Cell proliferation was carried out using the Cell Counting Kit-8 (Dojindo, Japan) based on the scheme provided by the manufacturer. Absorbance at OD 450 was calculated using an enzyme marker.

### Migration and invasion assays

Cell migration and invasion were performed according to the principles and steps of the Transwell assay.

### Dual-luciferase reporter gene assay

Wild-type (WT) and mutant (MUT) miR-16-5p 3'-UTR bearing the Linc00662 combining site were clocked in a pmirGLO vector (Promega, Madison, WI, USA). Post-transfection was assessed using a dual luciferase reporter assay system.

### Statistical analyses

All data are presented as mean ± SD. Prognostic values were analyzed using Kaplan–Meier and Cox regression with SPSS 21.0. The statistical characteristics of the patients were evaluated using the chi-square test. One-way ANOVA was visualized using Graphpad Prism 8.0. Data were judged as significant at *P* < 0.05.

## Results

### Expression of Linc00662 in CC

**Fig. 1:**
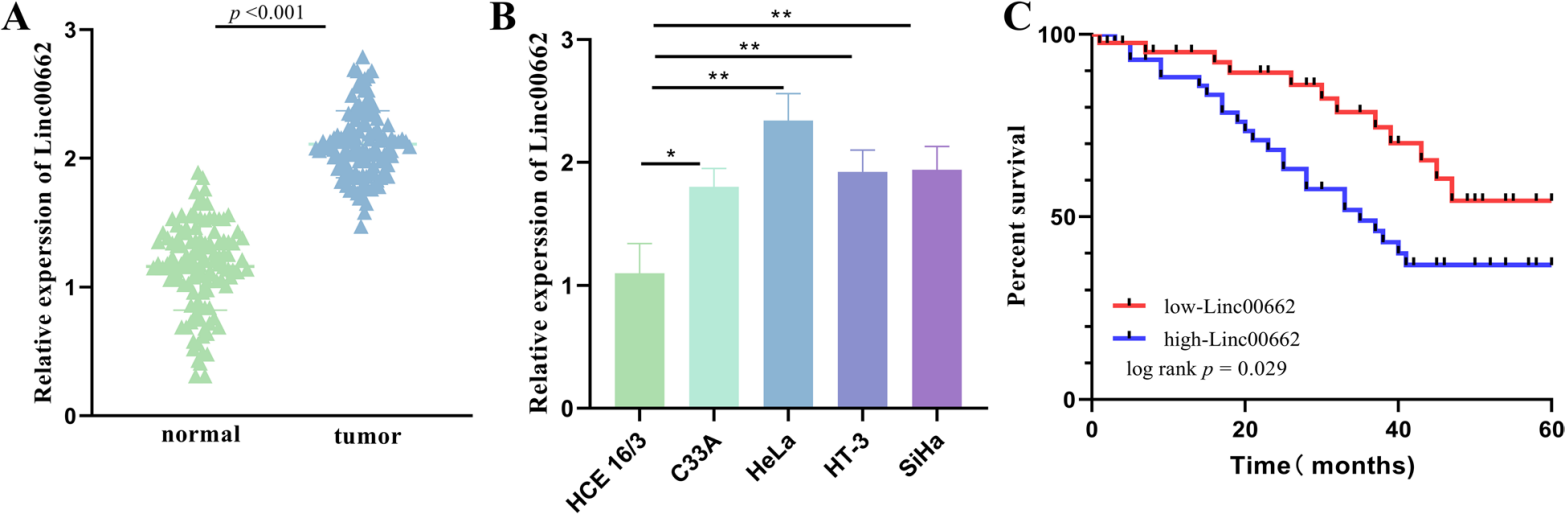
Linc00662 expression in CC. **A** Linc00662 expression in normal cervix and CC tissues. **B** Linc00662 expression levels in normal cervical epithelium and CC cell lines. **C** Correlation between survival and prognosis of CC patients based on Linc00662 expression

The expression of Linc000662 is higher in CC tissue than in the normal cervical tissues (Fig. 1A). The expression of Linc00662 was higher in CC cells relative to normal cervical epithelial cells (HCE 16/3) (Fig. 1B). Kaplan–Meier analyses suggested that patients with high Linc00662 expression had a markedly lower global survival rate, which portended a poor prognosis (Fig. 1C). Patients were grouped using the median value of Linc00662 expression level in CC as a cut-off point. The findings suggested that high expression of Linc00662 was linked to larger tumors (*P* = 0.006), later FIGO stage (*P* = 0.015), poorer tumor differentiation (*P* = 0.046), stromal infiltration (*P* = 0.002) and lymph node metastasis (*P* = 0.029) (Table 1). Cox proportional risk regression results showed that Linc00662 (HR = 11.875, 95% CI 3.068–45.965) was established as an independent prognostic indicator along with tumor size (HR = 0.188, 95% CI 0.05–0.7), FIGO stage (HR = 0.186, 95% CI 0.038–0.902), and lymph node metastasis (HR = 0.025, 95% CI 0.003–0.216) were established together as independent prognostic indicators (Table 2).

**Table 1:** Association between Linc00662 expression levels and clinical characteristics of patients with CC

Characteristic	Cases (*n* = 118)	Linc00662		*P*
		Low (*n* = 61)	High (*n* = 57)	
Age				0.985
< 45	56	29	27	
≥ 45	62	32	30	
Tumor size				0.006
< 4	46	31	15	
≥ 4	72	30	42	
FIGO stage				0.015
I	55	35	20	
II	63	26	37	
Differentiation				0.046
Poor	65	39	26	
Well or moderate	53	22	31	
Histology				0.735
Squamous	64	34	30	
Adenocarcinoma	54	27	27	
Stromal infiltration				0.002
No	57	38	19	
Yes	61	23	38	
Lymph node metastasis				0.029
Negative	50	20	30	
Positive	68	41	27	

*FIGO* The International Federation of Gynaecology and Obstetrics

**Table 2:** Multivariate logistic regression analyses of factors associated with CC

Variables	HR factor	95% CI	*P*- value
Linc00662	11.875	3.068–45.965	< 0.001
Age	6.20	0.016–23.784	0.797
Tumor size	0.188	0.05–0.7	0.013
FIGO stage	0.186	0.038–0.902	0.037
Differentiation	0.498	0.018–3.079	0.454
Histology	0.185	0.008–4.352	0.295
Stromal infiltration	0.206	0.006–6.99	0.380
Lymph node metastasis	0.025	0.003–0.216	< 0.001

*HR* Hazard ratio, *95% CI* 95% confidence interval, *FIGO* The International Federation of Gynaecology and Obstetrics

### Effect of Linc00662 on CC cell proliferation, migration and invasion

**Fig. 2:**
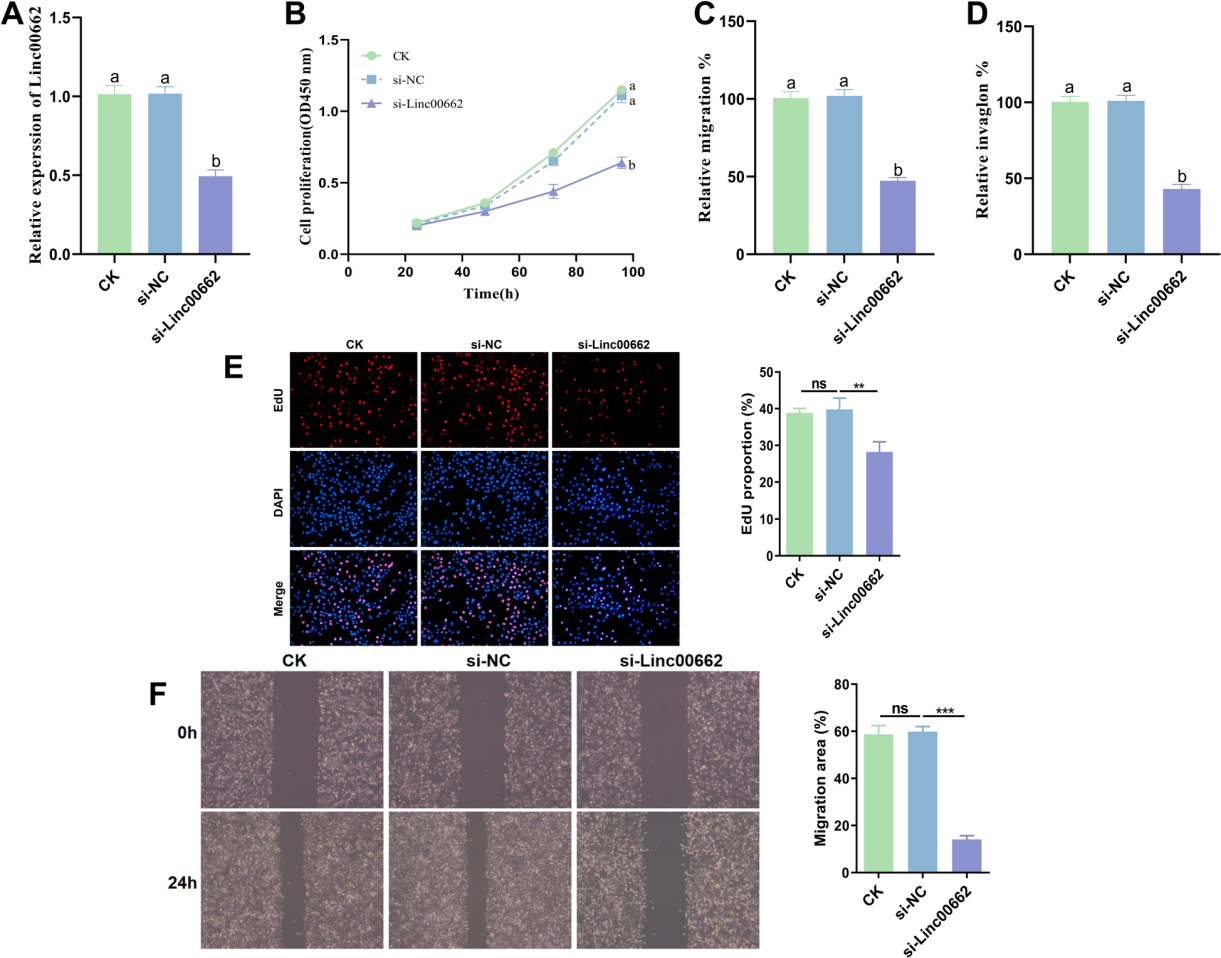
Effect of Linc00662 on proliferation, migration and invasion of CC cells. **A** Linc00662 expression level in HeLa cells. **B** Proliferation of HeLa cells. migration (**C**) and invasion (**D**) of HeLa cells. **E** EdU measurement (**F**) Wound healing assessment

Among the four types of CC cells, the highest level of Linc00662 expression was found in HeLa cells, so HeLa cells were used to silence Linc00662. The results suggested that silence Linc00662 caused a marked decrease in Linc00662 expression in HeLa cells compared with the control group (Fig. 2A). Silence Linc00662 significantly reduced the proliferation, migration and invasion of HeLa cells (Fig. 2B, C and D). The EdU results indicated that following the knockdown of Linc00662, the proportion of EdU-positive cells decreased (Fig. 2E). Additionally, the results of the wound healing assay demonstrated that the knockdown of Linc00662 inhibited cell migration ability (Fig. 2F).

### Linc00662 targets miR-16-5p

**Fig. 3:**
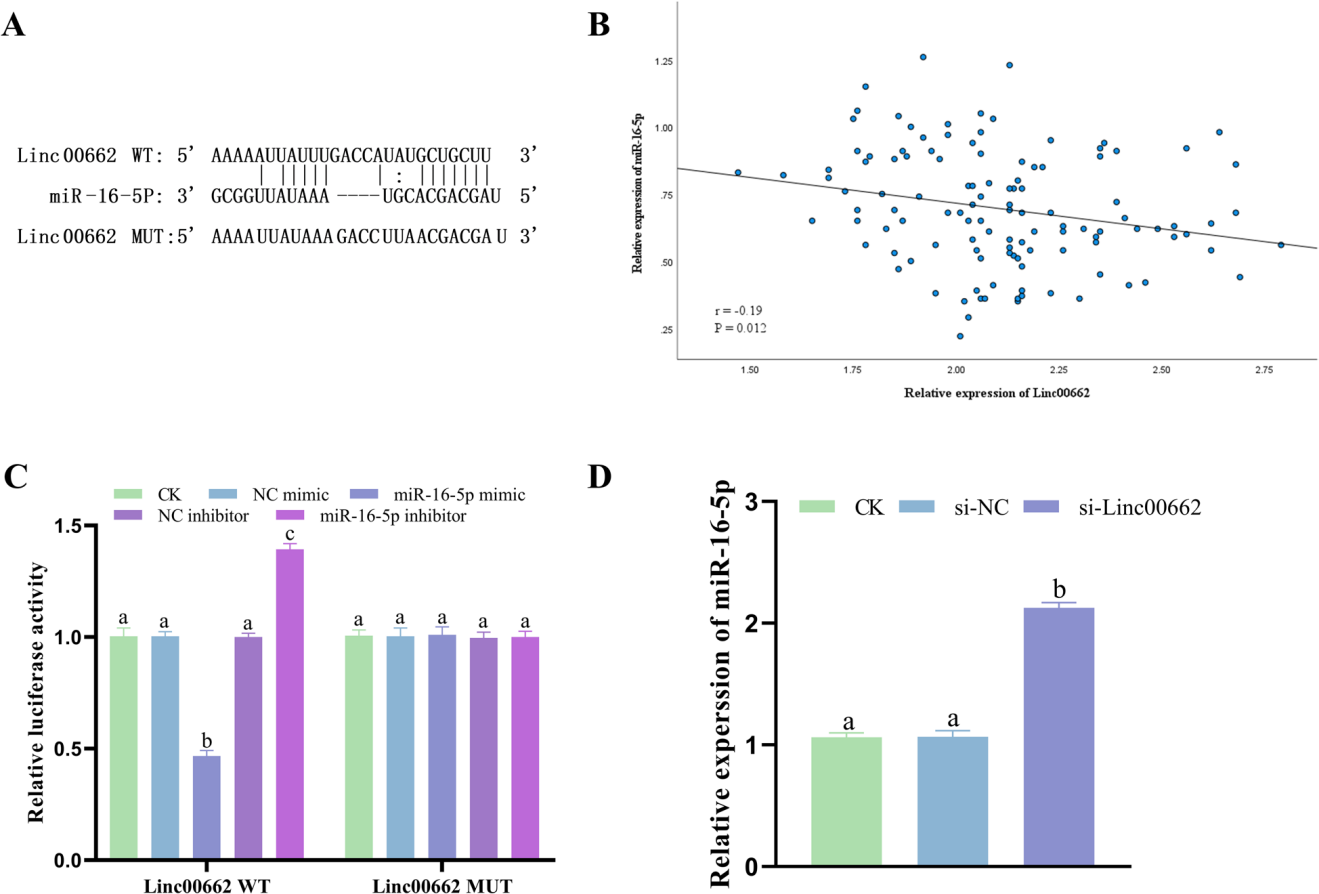
Linc00662 targets miR-16-5p. **A** Binding site between Linc00662 and miR-16-5p. **B** Linc00662 and miR-16-5p Pearson correlation analysis. **C** Dual luciferase reporter gene assay. **D** Expression of miR-16-5p in CC cells after Linc00662 silencing

The potential combining site between Linc00662 and miR-16-5p was identified by predicting the target miRNA of Linc00662 through the StarBase database (Fig. 3A). Pearson analysis suggested that Linc00662 was negatively linked to miR-16-5p (Fig. 3B, r = -0.19). Dual luciferase reporter gene assay showed that miR-16-5p mimics significantly reduced wild-type Linc00662 luciferase activity, whereas miR-16-5p inhibitors reversed this inhibition (Fig. 3C). Linc00662 silencing in HeLa cells significantly increased the expression level of miR-16-5p compared to controls (Fig. 3D).

### miR-16-5p expression in CC

**Fig. 4:**
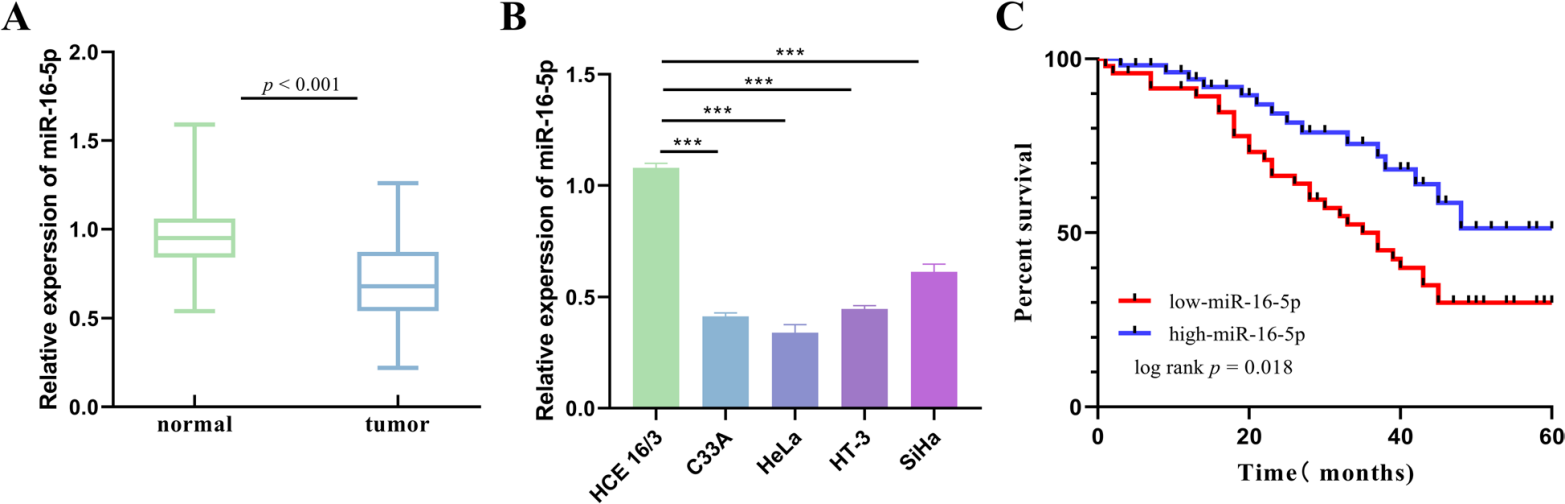
miR-16-5p expression in CC. **A** miR-16-5p expression in normal cervix and CC tissues. **B** miR-16-5p expression levels in normal cervical epithelium and CC cells. **C** Correlation between survival and prognosis of CC patients based on miR-16-5p expression

The expression of miR-16-5p was downregulated in CC tissues with respect to normal cervical tissues (Fig. 4A). miR-16-5p expression was also significantly downregulated in CC cells compared to normal cervical epithelial cells (HCE 16/3) (Fig. 4B). The results of the Kaplan–Meier survival analysis showed that patients with low miR-16-5p expression had significantly shorter global survival compared to patients with high miR-16-5p expression, which predicted a poor prognosis (Fig. 4C).

### Influence of miR-16-5p on CC cell proliferation, migration and invasion

**Fig. 5:**
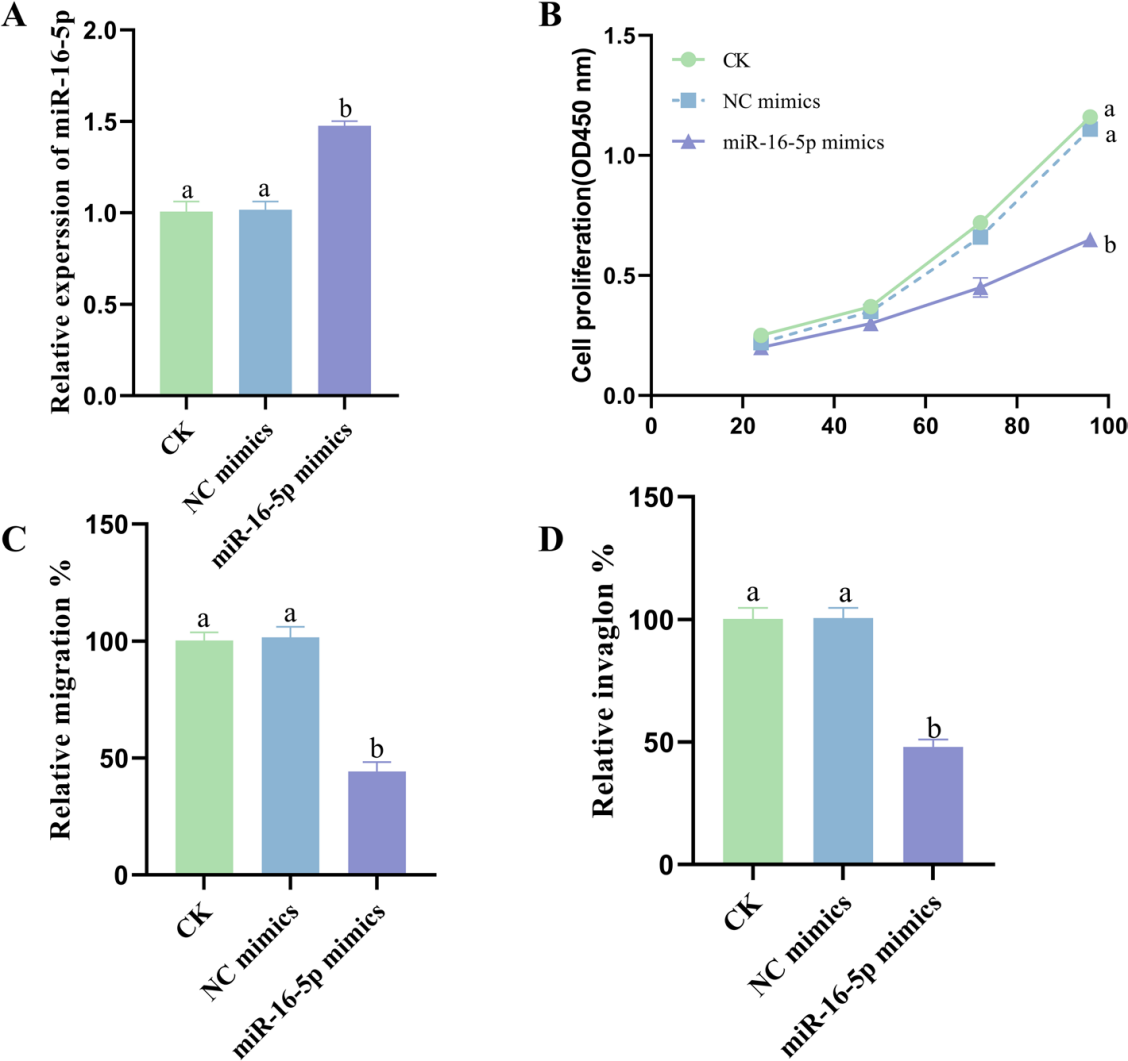
Influence of miR-16-5p on CC cell proliferation, migration and invasion. **A** miR-16-5p expression levels in HeLa cells. **B** Proliferation of HeLa cells. migration (**C**) and invasion (**D**) of HeLa cells

Among the four types of CC cells, HeLa cells had the lowest level of miR-16-5p expression, suitable for miR-16-5p overexpression. The results showed that miR-16-5p overexpression resulted in significantly higher expression in HeLa cells relative to the control group (Fig. 5A). miR-16-5p overexpression significantly inhibited the proliferation, migration and invasion of HeLa cells (Fig. 5B, C and D).

### FASN expression in CC

**Fig. 6:**
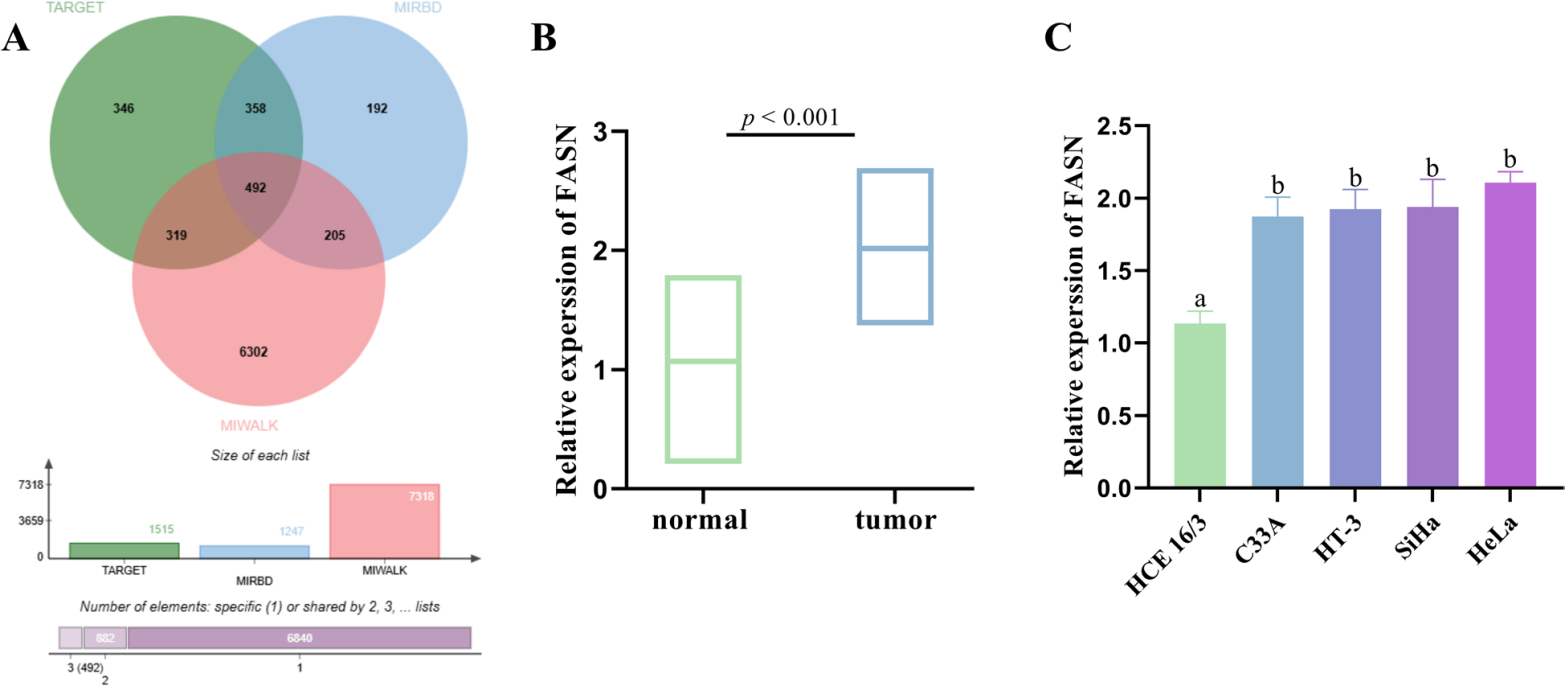
FASN is highly expressed in CC. **A** miR-16-5p target gene prediction. **B** FASN expression in normal cervical and CC tissues. **C** FASN expression levels in normal cervical epithelium and CC cells

Forecasting miR-16-5p downstream targets using TargetScan, miRDB and miRWalk databases to identify DEmRNAs with miR-16-5p bound loci (Fig. 6A). FASN exhibited remarkably higher expression in CC tissues when relative to normal cervical tissues (Fig. 6B). FASN expression was also clearly increased in C33A, HeLa, HT-3 and SiHa cells compared to normal cervical epithelial cells (HCE 16/3) (Fig. 6C).

### Regulation of FASN by Linc00662/miR-16-5p

**Fig. 7:**
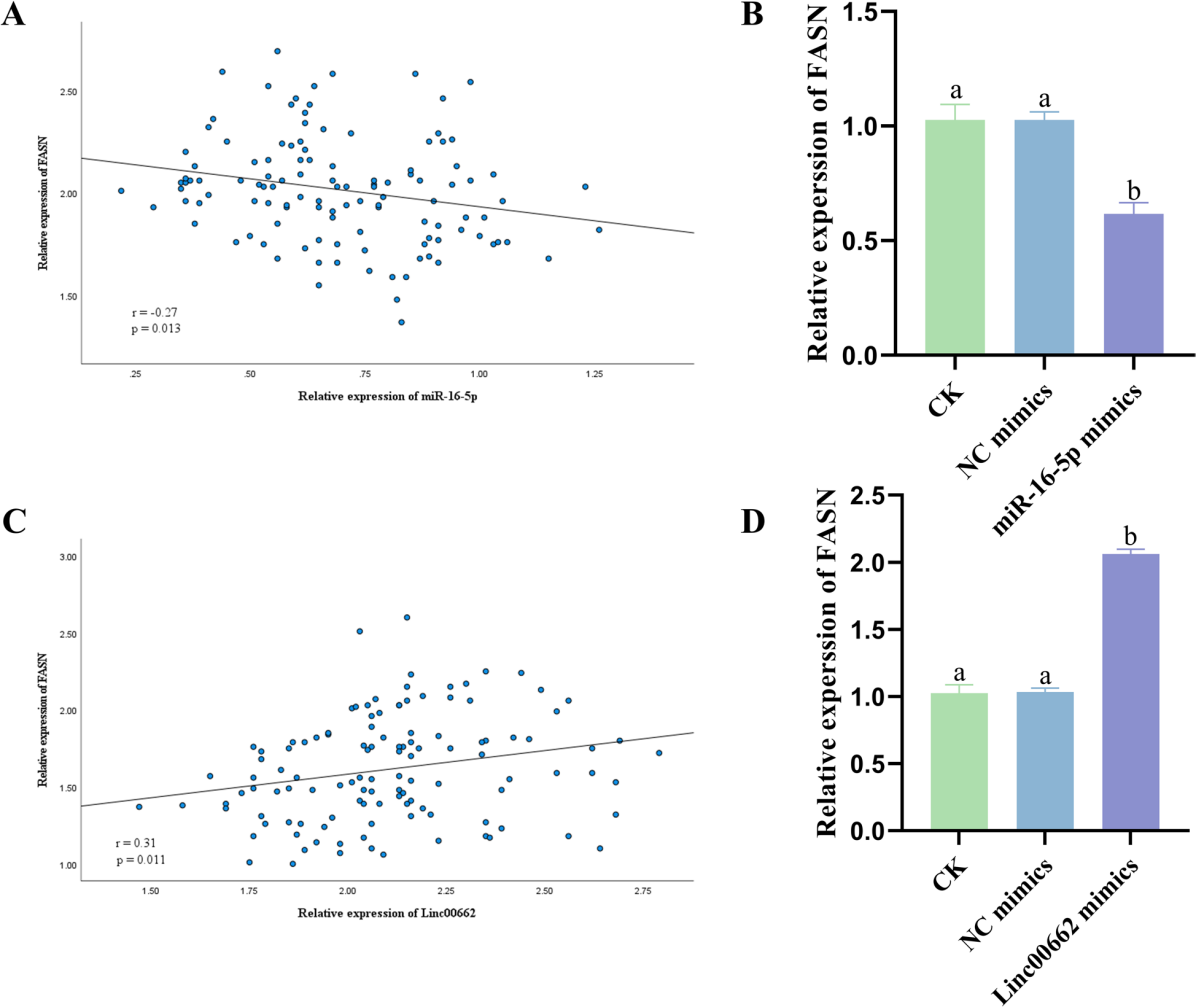
Regulation of FASN by Linc00662/miR-16-5p. **A** miR-16-5p and FASN Pearson correlation analysis. **B** Effect of miR-16-5p overexpression on FASN expression in CC. **C** Linc00662 and FASN Pearson correlation analysis. **D** Effect of Linc00662 overexpression on FASN expression in CC

Pearson analysis suggested that FASN was negatively linked to miR-16-5p (Fig. 7A, r = -0.27), while FASN was positively correlated with Linc00662 (Fig. 7C, r = 0.31). Among the four types of CC cells, the C33A cells had the lowest level of FASN expression, applicable to miR-16-5p overexpression. Overexpression of miR-16-5p led to a significant reduction in FASN expression in CC compared to control (Fig. 7B), while overexpression of Linc00662 caused a marked rise in FASN expression in CC (Fig. 7D).

### Reversal effect of miR-16-5p

**Fig. 8:**
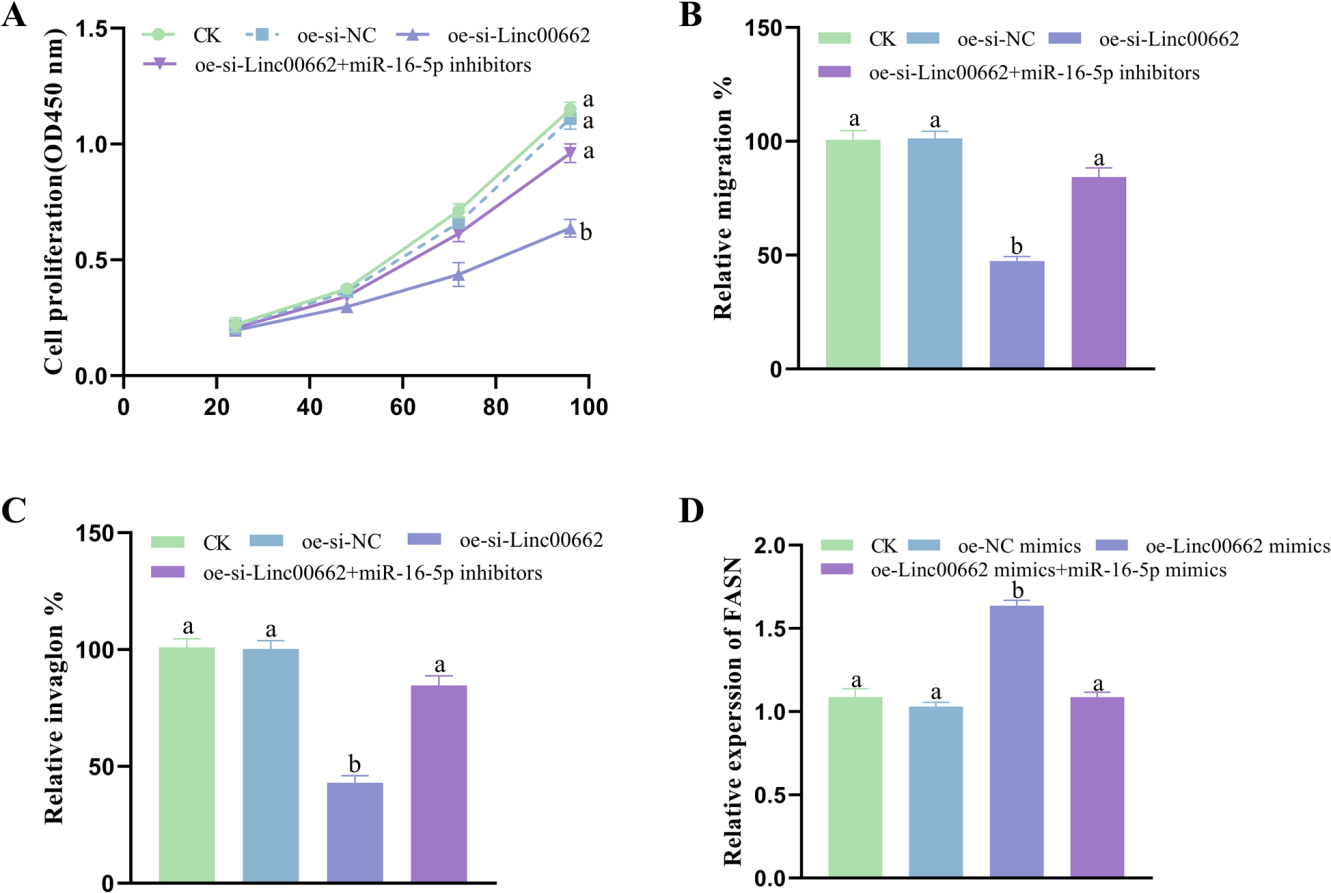
Reversal effect of miR-16-5p. **A** Proliferation of CC cells. **B** Migration of CC cells. **C** Invasion of CC cells. **D** Expression level of FASN in CC cells

To elucidate further the effects of Linc00662/ miR-16-5p/FASN in CC, miR-16-5p inhibitors were transfected into Linc00662-silenced HeLa cells. The findings suggest that miR-16-5p inhibitor was able to significantly reverse the Linc00662 silencing-mediated inhibition of CC cell proliferation (Fig. 8A), migration (Fig. 8B) and invasion (Fig. 8C). Furthermore, miR-16-5p mimics were transfected into Linc00662 overexpressing C33A cells. The obtained results displayed that miR-16-5p mimic reversed the Linc00662 overexpression-induced up-regulation of FASN expression level (Fig. 8D).

## Discussion

Recent lncRNA studies have revealed key regulators of CC progression, such as upregulation of LncRNA PCGEM1 expression in CC cells, which promotes CC progression through the miR-642a-5p/KIF5B axis [26]; LncRNA LINC00649 expression is increased in CC tissues, and LINC00649 expression serves as a prognostic predictor and exacerbates cervical cancer progression by targeting miR-216a-3p [27]; FOXD2-AS1 enhances the growth, invasion, and migration of oral squamous cell carcinomas by modulating the miR-185-5p/PLOD1/Akt/mTOR signaling pathway [28]. Linc00662 plays a key role in some cancers. Linc00662 promotes breast cancer cell viability and reduces apoptosis [29]. Linc00662 facilitates hepatocellular carcinoma advancement by regulating a genomic methylation [30]. In CC, Linc00662 accelerates CC cell proliferation and invasion and suppresses apoptosis through the upregulation of PDK4 [23]. In this work, the expression level of Linc00662 was higher in CC, and its high expression predicted a poor prognosis. Linc00662, tumor size, FIGO stage, and lymph node metastasis were established together as independent prognostic indicators. Silencing Linc00662 reduced the proliferation, migration, and invasion of CC cells. In summary, Linc00662 plays a key role in promoting CC advancement.

miRNAs are vital regulators in cancer. miR-16-5p inhibits proliferation, migration and invasion of breast cancer cells and suppresses breast cancer progression by targeting ANLN [31]. miR-16-5p expression is significantly down-regulated in osteosarcoma and suppresses osteosarcoma progression and invasion by targeting Smad3 [32]. The findings suggested that miR-16-5p expression levels were lowered in CC tissues and cells; low miR-16-5p expression predicted a poor prognosis; and miR-16-5p overexpression inhibited the proliferation, migration and invasion of HeLa cells. This is consistent with previous findings. LncRNAs can directly interplay with miRNAs to modulate cancer progression [33]. This mechanism is gaining importance and attention in cancer research. LncRNA DUXAP8 enhances renal cell carcinoma progression by negatively regulating miR-126 [34]. In this work, we ascertained the combining site of Linc00662 to miR-16-5p and miR-16-5p was negatively regulated by Linc00662 in CC cells.

Lipid metabolism is recognized as a crucial pathway in cancer, with FASN serving as the central regulatory factor. FASN plays a vital role in tumor growth and survival, demonstrating significant potential for clinical applications [35]. Fatty acid-binding protein 5 regulates the expression of FASN through the ubiquitin–proteasome pathway, activates the WNT/β-catenin signaling pathway, and modulates lipid metabolism to promote the progression of pancreatic neuroendocrine tumors [36]. FTO-induced APOE activates the PI3K/AKT/mTOR signaling pathway by regulating the ubiquitination state of FASN, which enhances lipid metabolism and proliferative capacity, thereby promoting the malignant progression of pancreatic neuroendocrine tumors [37]. FASN expression is upregulated in CC tissues and promotes CC cell migration and invasion through cholesterol reprogramming, lymph node metastasis in CC [38]. The EGF-CSN6-FASN axis promotes colorectal cancer growth [18]. In this work, we identified the target of miR-16-5p, and the analysis suggested that the expression of FASN was markedly elevated in CC tissues and cells. FASN was negatively linked to miR-16-5p and positively linked to Linc00662. Linc00662 may enhance the expression of FASN by down-regulating miR-16-5p. In addition, other lncRNA-miRNA-mRNA axes also mediate the progression of CC. For example, lncRNA DLG1-AS1 enhances the resistance of cervical cancer cells to gemcitabine by regulating the miR-16-5p/HDGF pathway [39]. Furthermore, lncRNA HOTAIR contributes to carcinogenesis by targeting the miR-331-3p/RCC2 axis [40].

From the perspective of traditional medicine, the integration of network pharmacology analysis with disease targets provides novel insights for disease research. For instance, Xu et al. identified AKT1, MAPK1, MYC, EGF, and HSP90AA1 as key targets for treating Major Depressive Disorder with Hypericum perforatum [41]. Additionally, Liu HR utilizes traditional medicine in conjunction with biomarker-driven methods to mitigate the progression of esophageal cancer induced by Trichostatin A [42]. Linc00662, as a novel drug target, requires further research and exploration regarding its role in regulating tumor progression and treatment. Research has demonstrated that pan-cancer analysis of copper toxicity genes and gene sets related to copper metabolism can elucidate their effects on the cancer microenvironment and drug resistance. This approach offers valuable insights for cancer diagnosis, prognosis, and the development of novel clinical treatments [43, 44]. Additional research is required to analyze the entire cancer gene network in CC.

## Conclusion

The findings of this work suggest that Linc00662 positively regulates FASN expression through targeting miR-16-5p and promotes CC cell proliferation, migration and invasion, which in turn facilitates CC progression. Therefore, Linc00662 has to be a therapeutic target and a prognostic biomarker for patients with CC.

## Data Availability

No datasets were generated or analysed during the current study.
